# Case report: Intracranial and spinal subarachnoid hemorrhage in a dog with Angiostrongylosis

**DOI:** 10.3389/fvets.2023.1190792

**Published:** 2023-05-24

**Authors:** Koen M. Santifort, Marjolein den Toom, Laurent Garosi, Ines Carrera

**Affiliations:** ^1^IVC Evidensia Small Animal Referral Hospital Arnhem, Neurology, Arnhem, Netherlands; ^2^IVC Evidensia Small Animal Referral Hospital Hart van Brabant, Neurology, Waalwijk, Netherlands; ^3^IVC Evidensia Small Animal Referral Hospital Nieuwegein, Cardiology, Nieuwegein, Netherlands; ^4^Vet Oracle Teleradiology, Norfolk, United Kingdom

**Keywords:** T1-hyperintense, *Angiostrongylus vasorum*, hyperesthesia, canine (dog), subarachnoid hemorrhage

## Abstract

A 1-year-old male intact Staffordshire terrier, born and raised in the Netherlands, was presented with a 3-week history of progressive lethargy and spinal, predominantly cervical, hyperesthesia. Other than hyperthermia and cervical hyperesthesia, general and neurological examination did not reveal any abnormalities. Comprehensive hematological and biochemical tests were considered normal. Magnetic resonance imaging of the craniocervical region revealed heterogeneity of the subarachnoid space, characterized by pre-contrast T1W hyperintensity, corresponding to a T2* signal void. Extending from the caudal cranial fossa to the level of the third thoracic vertebra, there were uneven patchy extra-parenchymal lesions that caused mild spinal cord compression, most marked at the level of C2. At this level, the spinal cord showed an ill-defined hyperintense T2W intramedullary lesion. Mild intracranial and spinal meningeal contrast enhancement was evident on post-contrast T1W images. Subarachnoid hemorrhage was suspected, and further diagnostic tests including Baermann coprology resulted in a diagnosis of hemorrhagic diathesis caused by an *Angiostrongylus vasorum* infection. The dog rapidly responded to treatment with corticosteroids, analgesic medication, and antiparasitic treatment. Follow-up over 6 months yielded complete clinical remission and repeatedly negative Baermann tests. This case report details clinical and magnetic resonance imaging findings in a dog with subarachnoid hemorrhage associated with an *Angiostrongylus vasorum* infection.

## Introduction

Central nervous system (CNS) extra-parenchymal hemorrhages can be located within the epidural, subdural, subarachnoid (SAH), and intraventricular spaces (1, 2). Intraparenchymal CNS hemorrhage is more common in dogs than extra-parenchymal hemorrhage and is often caused by traumatic brain injury, vascular disease, neoplasia, or infection (1, 2). One major difference in intraparenchymal CNS hemorrhage in dogs is an infection with *Angiostrongylus vasorum* (*A. vasorum*) (3–5). Subarachnoid hemorrhage has not been diagnosed clinically in dogs with *A. vasorum* infections. This case report details clinical and magnetic resonance imaging findings in a dog with subarachnoid hemorrhage caused by an *A. vasorum* infection.

### Case description

A 1-year-old male intact 18 kg Staffordshire terrier, born and raised in the Netherlands, was presented with a 1-week history of progressive lethargy and spinal hyperesthesia. The vaccination regimen was followed according to regional guidelines, but the dog received no antiparasitic treatment. The dog was kept in a suburban environment and occasionally went to woodlands for walks. The history provided by the referring veterinarian documented multiple occasions in which the dog had a high rectal temperature (ranging from 39.3 to 39.9°C). Treatment with meloxicam (0.1 mg/kg q24h) and gabapentin (5–10 mg/kg q8-12h) had not resulted in remission of clinical signs. General clinical examination upon presentation to the referral hospital revealed a rectal temperature of 39.8°C. The dog was panting and breathing heavily, but thoracic auscultation was deemed unremarkable. There were no signs of hemorrhage in any mucous membranes or sclera at this point in time. Neurological examination revealed a low-head carriage and guarded posture, with severe cervical muscular hypertonia and resistance to passive movement of the neck consistent with cervical hyperesthesia. Hyperesthesia was also evident upon palpation of the thoracic and lumbar (para)spinal regions, but to a lesser degree. Occasional vocalization was noticed during the examination and when the dog stepped off ledges.

Preanesthetic hematological and biochemical blood tests did not reveal any abnormalities, including C-reactive protein (CRP; <1 mg/L, with reference range 0.0–10.0). The dog was anesthetized, and a magnetic resonance imaging (MRI, 1.5 T Canon Vantage Elan) study of the cervical region including part of the head up to the cranial thorax was performed including the following sequences: sagittal and transverse T2-weighted spin echo (T2W), T1- weighted spin echo (T1W), sagittal short tau inversion recovery (STIR), and transverse T2* gradient echo (T2*). Transverse and sagittal T1-weighted images were also acquired after contrast-medium administration together with transverse fat saturation images.

**Figure 1:**
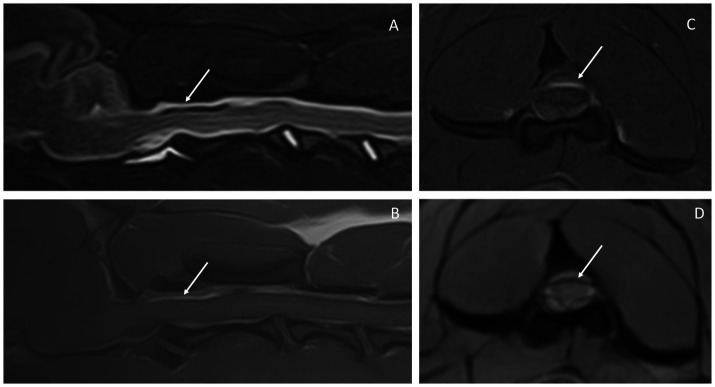
Magnetic resonance images of the reported case. Sagittal STIR **(A)**, sagittal T1W pre-contrast **(B)**, transverse T1W FATSAT post-contrast **(C)**, and transverse T2* **(D)** at the level of C2. The arrows point out the subarachnoid space, which is heterogeneous in all sequences, showing mixed signal intensity on STIR **(A)** and hyperintensity in T1W **(B)**. The latter is seen clearly in **C**, in which the epidural fat is suppressed and the hyperintensity within the subarachnoid space is highlighted. This same area corresponds to signal void in T2* **(D)**.

The MRI study of the cervical spine and part of the cranium showed a diffuse abnormal signal intensity of the subarachnoid space, characterized by patchy T2W and STIR hypointensities, which corresponded to T1W pre-contrast hyperintensities and linear T2* signal voids (Figure 1). This intradural/extra-parenchymal lesion extended from the caudal fossa to at least the level of T3. The lesion caused the subarachnoid space to be irregular and uneven in appearance with associated spinal cord compression, that was most evident at the level of C2. At this level, the spinal cord showed a mild increase in T2W signal intensity, which was isointense in T1W and T2* hyperintense. From the post-contrast images, mild meningeal enhancement could be appreciated within the caudal fossa and the cranial cervical spine. No intraparenchymal contrast enhancement was seen.

The diffuse abnormal signal intensity of the cervical subarachnoid space was considered compatible with a subarachnoid hemorrhage based on the T2* signal void and T1W pre-contrast hyperintense signal. Differential diagnoses considered at that time included steroid responsive meningitis arteritis (SRMA), bacterial meningitis, and infectious disorders with a concurrent hemorrhagic diathesis. Due to concerns about a hemorrhagic diathesis, a cisternal cerebrospinal fluid (CSF) tap was deemed to be a high risk; a single attempt at a lumbar CSF tap between L5 and L6 resulted in a subcutaneous hematoma prior to entering the vertebral canal, and the attempt was abandoned. The dog was recovered from anesthesia and admitted for treatment, monitoring, and further diagnostic testing.

Treatment was initiated with dexamethasone 0.2 mg/kg IV once, followed by 0.5 mg/kg prednisolone q12h, ketamine 5 ug/kg/min continuous rate infusion IV, amoxicillin/clavulanic acid 20 mg/kg q12h IV (discontinued after 36 h), methadone IV based on pain scores (0.3–0.5 mg/kg), and paracetamol 12 mg/kg q8h IV. An increased dosage of prednisolone for the differential diagnosis of SRMA was considered, but the dog showed a rapid response to the treatment outlined above, exhibiting markedly improved mobility and head carriage within a few hours though still having a guarded posture and seemingly fearful to be touched. The latter remitted within 48 h, during which further diagnostic tests and repeated clinical examinations were performed, yielding more information not compatible with a diagnosis of SRMA.

Of the further diagnostic tests, the following were all within the reference range or interpreted as “normal” or negative: serology for *Toxoplasma gondii* (IgM and IgG), *Neospora caninum*, and *A. vasorum* (Angio Detect™, IDEXX The Netherlands), SNAP test for *Ehrlichia canis*, *Dirofilaria immitis*, *Anaplasma phagocytophilum*, and *Borrelia burgdorferi* (SNAP 4Dx Plus, IDEXX The Netherlands), urinalysis, bacteriological urine culture and blood culture, and a coagulation profile (including activated partial thromboplastin time (aPTT), partial thromboplastin time (PT), fibrinogen, thrombin time (coagulometry), von Willebrand’s factor, and antithrombin III (chromogenic assay)).

Buccal mucosal bleeding time (BMBT) was 7 min and deemed abnormal (reference range: <4 min). Blood pressure varied between measurements (with a range of 120–220 mmHg), but higher values were deemed to be the result of inadequate analgesia and stress. Reevaluation of thoracic auscultation (after recovery from anesthesia when the dog was not panting and had a normal respiratory rate and rhythm) revealed increased inspiratory sounds. Thoracic radiographs revealed moderate generalized increased pulmonary opacity, characterized by generalized bronchial thickening and blurring of the vascular detail, indicating a moderate broncho-interstitial lung pattern. No signs of air bronchograms and alveolar pattern neither pleural nor mediastinal disease were present.

The day after admission, the dog exhibited a scleral subconjunctival hematoma in the right eye and in the evening in the other eye as well. A rectally acquired fecal sample on the day of neurological examination showed some smears of fresh blood. A Baermann test (one-day fecal sample attained in hospital) was performed, and *A. vasorum* first-stage larvae were morphologically identified. Milbemycin oxime/praziquantel (12.5 mg/125 mg tablet, once weekly for 4 weeks; Milbemax ®, Elanco, the Netherlands) was added to the treatment. The dog was discharged after 72 h and prescribed a combination of paracetamol 12 mg/kg q8h *per os* and prednisolone 0.3 mg/kg q24h *per os*. After 4 days, the patient was represented and admitted due to relapse of signs of cervical hyperesthesia though subjectively mild compared to initial clinical signs. The dog was admitted for another 2 days, during which additional analgesia was provided with gabapentin 10 mg/kg q8h *per os* and prednisolone dosage was increased to 0.5 mg/kg q24h *per os*. Treatment was then continued at home, and tapering dosages of gabapentin (10 mg/kg q8h for 7 days, followed by 10 mg/kg q12h for 7 days, followed by 5 mg/kg q24h for 7 days), paracetamol (12 mg/kg q12h for 7 days), and prednisolone (0.5 mg/kg q24h for 6 weeks, followed by 0.4 mg/kg q24h for 2 weeks, followed by 0.3 mg/kg q24h for 2 weeks, followed by 0.3 mg/kg q48h for 4 weeks and 0.1 mg/kg q48h for 4 weeks).

Over the next 6 months, monthly revisits were scheduled during which clinical examinations as well as Baermann tests (3-day fecal samples) and thoracic radiographs were repeated. The Baermann test remained positive 1 month (34 days) after starting treatment, and antiparasitic treatment was switched to imidacloprid/moxidectin (250 mg/62.5 mg spot on, monthly; Advocate ®, Elanco, the Netherlands). Baermann coprology was negative at 2 months, 3 months, and 6 months after discharge. Thoracic radiographs at 1, 2, and 4 months showed minimal changes from the first study. Over time, the diffuse interstitial pattern was unchanged and a slight improvement in the bronchial thickening was appreciated. However, the dog did not show any signs of exercise intolerance (able to run and play for over an hour). Lifelong treatment with imidacloprid/moxidectin (250 mg/62.5 mg spot on, monthly; Advocate ®, Elanco, the Netherlands) was recommended to prevent reinfection with *A. vasorum*.

## Discussion

This is the first case report describing SAH in a dog with an *A. vasorum* infection. Confirmation of SAH can be clinically achieved by acquiring a CSF sample from a patient which contains red blood cells (RBCs) (*caveat* contamination by puncture of a blood vessel) and in which the suspicion was further substantiated by clinical and imaging findings (6, 7). In cases where there is a possible contraindication for performing a CSF tap (e.g., hemorrhagic diathesis) and a clinical suspicion of SAH, the lack of a CSF tap hampers a definitive diagnosis. Still, MRI findings coupled with clinical and diagnostic test results can be used to substantiate the diagnosis. Due to the value of confirming the suspected diagnosis of SAH in this case and uncertainty pertaining to the presence or not of hemorrhagic diathesis (as a contraindication for a CSF tap), a lumbar CSF tap was performed. However, the attempt was quickly abandoned when subcutaneous hematoma further substantiated suspicions of hemorrhagic diathesis. In a recent study on complications associated with CSF collection in 102 dogs, subcutaneous hematoma formation was documented as a minor complication in 10 dogs (9.8%) (8). None of those 10 dogs were suspected or proven to have a coagulopathy, however. Indeed, CSF collection was not attempted in 1 dog in that study where coagulopathy was suspected. In the canine case reported here, there are several reasons why there is little room for doubt as to the diagnosis of SAH: (i) T1-hyperintensity in the subarachnoid space on MRI; (ii) clinical evidence of hemorrhagic diathesis (scleral hemorrhages and BMBT results); (iii) a plausible cause for hemorrhagic diathesis (*A. vasorum* infection); and (iv) resolution of clinical signs after treatment.

Intraspinal hemorrhages are classified according to their location as extradural hematoma, subdural hematoma, hematomyelia, and subarachnoid hemorrhage (9). In people, the main causes for SAH without preceding trauma include rupture of an aneurysm, other vascular malformations, and vasculitis (10); aneurysms are the main cause of SAH reported in humans (11). Spinal aneurisms and vascular malformations such as arteriovenous malformations in dogs are uncommon, and only a few case reports have been published (12–17). However, SAH has not been reported in combination with these kinds of vascular spinal malformations in dogs. A common feature of aneurysms and vascular malformations is the presence of a fairly well-defined lesion which causes a mass effect (11–17). This was lacking in MR images of the case reported here. Therefore, these differentials were excluded. In the canine case reported here, *A. vasorum* infection was considered a plausible cause underlying the SAH. Other causes for extra-parenchymal spinal cord hemorrhage (including SAH as well as epidural hemorrhage) or hematoma in dogs that have been reported, include SRMA, intervertebral disc disease, neoplasia, and snake envenomation (18–20). In human medicine, several other differential diagnoses are reported including bacterial meningitis (21). This last differential was briefly considered in our case which was the reason why bacteriological urine culture and blood culture were performed and antibiotic treatment was initiated. The latter was discontinued as soon as other diagnostic test results and clinical findings suggested much more plausible explanations. Theoretically, all disorders causing vasculitis or coagulopathy could result in SAH. When no cause is identified, the term “spontaneous hemorrhage or hematoma” is used (18). Of these differential diagnoses, SRMA was considered prominently in our case and is discussed in more detail here.

SRMA was considered a likely differential diagnosis on the day of admission after obtaining the MRI study based on an earlier report documenting similar findings (21), signalment, and clinical findings. Cervical hyperesthesia in dogs can be caused by a variety of disorders affecting skin, muscles, joints, bones, ligaments, meninges, nerves, nerve roots, spinal ganglia, intervertebral discs, or may indeed be caused by pathology at other sites (e.g., “thalamic pain”). Recent studies focusing on canine cervical hyperesthesia have determined intervertebral disc disease and SRMA to be the most common causes of cervical hyperesthesia (22, 23). A clinical cervical localization is more likely in older dogs with specific clinical signs (suggestive of a focal lesion, e.g., intervertebral disc extrusion) compared to a multifocal localization, which is more likely in younger dogs (23). Response to treatment including corticosteroids (dexamethasone followed by prednisolone) can be considered consistent with SRMA. However, in the case reported here, it was considered incompatible that CRP levels were < 1 mg/L as CRP is regarded as a sensitive (though not specific) biomarker for SRMA (24). The dosages included in the treatment of this case were below that typically used for treatment regimens in SRMA. Despite that, the dog responded rapidly to the treatment that was initiated. The clinical findings of scleral subconjunctival hematoma formation the next day and results of further diagnostic testing did not support a diagnosis of SRMA. All three dogs with neurological signs included in a recent study on the coagulation status of dogs naturally infected with *A. vasorum* all presented with episcleral bleeding (25). Authors of that publication commented that this finding should be investigated in a larger study population, supported by the case reported here. The decision for the fairly long-term application of prednisolone in this case was mainly based on the preference for slow tapering of the drug and the hypothesis that inflammatory reactions might result from the presence of blood components in the subarachnoid space contributing to cervical hyperesthesia. The authors would like to stress that there is no evidence to support the use of prednisolone for the management of either SAH or *A. vasorum* infections in dogs. This case report emphasizes that SAH should be included as a cause of spinal hyperesthesia in dogs.

Though SAH due to *A. vasorum* infection in dogs has not been previously reported, a major differential for intraparenchymal CNS hemorrhage in dogs is an infection with *A. vasorum* (3–5). The variability of reported MRI findings of such intraparenchymal CNS hemorrhage is largely due to the differences in timing of hemorrhagic events (1, 3–5). One study reported that the majority of the lesions were isointense or slightly hyperintense in T1-weighted images, hyperintense in T2-weighted images, and, most characteristically, hypointense in T2*-weighted (gradient echo) images (consistent with subacute-chronic hemorrhage) (26). The findings of SAH reported in this case were mostly different due to the localization of the signal abnormalities (i.e., subarachnoid space extraparenchymal localization), with findings of patchy hypointensities in T2W and STIR, which corresponded to hyperintensities in T1W pre-contrast and linear signal void areas from the T2* sequence. Some of these findings are mirrored in other reports on SAH in dogs with variable etiologies (18–20).

The pathogenesis of the hemorrhage due to *A. vasorum* infection is incompletely understood but does not seem to necessarily involve parasitic intraparenchymal invasion. Hemorrhagic diathesis is postulated to result from dysregulation of hemostatic proteins (5, 25). Results of coagulation tests in dogs with *A. vasorum* infections, such as BMBT, aPTT, and PT, are highly variable; one study reported no significant differences in PT and aPTT between dogs with and without hemorrhagic diathesis (27). Another recent study did find significant differences for some parameters including PT between hypocoagulable and normo-coagulable dogs with *A. vasorum* infections. It is considered prudent to perform coagulation tests in every case (5, 25). The value of some other coagulation tests, such as thromboelastometry (ROTEM), which were not performed in this case, are discussed in recent literature as well (25, 28).

Regarding the diagnosis of *A. vasorum* infections in dogs, the commercially available Angio Detect™ (IDEXX, The Netherlands) in the house test has been reported to have a high specificity (up to 100%) but a low to moderate sensitivity compared to Baermann coprology (29). This means that false-negative results can and do occur, as exemplified in the case reported here, where the Baermann test was positive and the Angio Detect™ test was negative. Regarding the value of radiography for the diagnostic work-up of dogs with *A. vasorum* infections, it is important to note that thoracic radiographs are recommended in essentially all (suspected) cases (30, 31). A mild to severe increase in density of interstitial and bronchial tissue may be apparent on thoracic radiographs (30, 31).

*A. vasorum* is endemic in the Netherlands in addition to numerous other Northern European countries (32–34). Clinical manifestation of *A. vasorum* infections in dogs can vary greatly and includes, but is not limited to, (cardio)respiratory, bleeding, and neurological disorders (30, 31, 35). For dogs with bleeding disorders, intracranial and spinal cord hemorrhage is one of the most commonly reported effects and can have a fatal outcome (3, 4, 26, 35–37). Treatment is aimed at supportive therapy and administration of anthelmintic drugs. Currently, there are two licensed anthelmintic treatments that include an indication for *A. vasorum* in their label claims in the Netherlands. The first is a milbemycin oxime tablet, in combination with praziquantel (Milbemax ®, Elanco The Netherlands). Milbemycin has been shown to be effective in reducing levels of adult and immature adult (L5) infection, and weekly oral administration for 4 weeks is required to treat clinical *A. vasorum* infection (38). The second is a moxidectin/imidacloprid spot-on solution (Advocate®, Elanco, the Netherlands), that requires a single monthly spot-on application to eliminate infection. Moxidectin has been shown to be effective in removing adult *A. vasorum* and immature stages (L4 and L5) (39, 40). In this case, the owner preferred treatment with tablets, and therefore, initially, milbemycin oxime/praziquantel tablets were chosen for treatment. After 4 weekly administrations, a repeat Baermann test (34 days after initial treatment) remained positive. It should be noted that this does not necessarily mean that the treatment with milbemycin containing tablets was not effective. *A. vasorum* L1 stages are not eliminated with the use of milbemycin, and it takes some time for L1 parasites to migrate out of the lungs. Additionally, adult worms may not die at the onset of treatment with antiparasitic drugs directly, and the shedding of L1 may be prolonged even further. Repeat Baermann tests 1, 2, and 5 months after starting imidacloprid/moxidectin spot on treatment were all negative.

In conclusion, we report a case of SAH in a dog infected with *A. vasorum*. Specific MRI findings were of great value to swiftly reach a diagnosis and perform additional relevant tests to find the underlying cause.

## Data availability statement

The original contributions presented in the study are included in the article/supplementary material, further inquiries can be directed to the corresponding author.

## Ethics statement

Ethical review and approval was not required for this case report since the animal was treated in accordance with the local legislation and institutional requirements. Written informed consent was obtained from the owners for the participation of their animal in this study.

## Author contributions

KS and MT contributed to the management of the case. KS, LG, and IC interpreted MRI findings. KS wrote the first draft of the manuscript. IC provided the figure for publication. KS, MT, LG, and IC participated in the revision of the manuscript. All authors read, commented on, and approved the final manuscript. All authors contributed to the article and approved the submitted version.

## Funding

The publication fee was covered by IVC Evidensia’s fund for publication of peer-reviewed scientific articles.

## Conflict of interest

The authors declare that the research was conducted in the absence of any commercial or financial relationships that could be construed as a potential conflict of interest.

## Publisher’s note

All claims expressed in this article are solely those of the authors and do not necessarily represent those of their affiliated organizations, or those of the publisher, the editors and the reviewers. Any product that may be evaluated in this article, or claim that may be made by its manufacturer, is not guaranteed or endorsed by the publisher.
